# Collinear Hox-Hox interactions are involved in patterning the vertebrate anteroposterior (A-P) axis

**DOI:** 10.1371/journal.pone.0175287

**Published:** 2017-04-11

**Authors:** Kongju Zhu, Herman P. Spaink, Antony J. Durston

**Affiliations:** Institute of Biology, Leiden University, Leiden, the Netherlands; University of Colorado Boulder, UNITED STATES

## Abstract

Investigating regulation and function of the Hox genes, key regulators of positional identity in the embryo, opened a new vista in developmental biology. One of their most striking features is collinearity: the temporal and spatial orders of expression of these clustered genes each match their 3’ to 5’ order on the chromosome. Despite recent progress, the mechanisms underlying collinearity are not understood. Here we show that ectopic expression of 4 different single Hox genes predictably induces and represses expression of others, leading to development of different predictable specific sections of the body axis. We use ectopic expression in wild-type and noggin—dorsalised (*Hox*-free) *Xenopus* embryos, to show that two Hox-Hox interactions are important. Posterior induction (induction of posterior Hox genes by anterior ones: PI), drives Hox temporal collinearity (Hox timer), which itself drives anteroposterior (A-P) patterning. Posterior prevalence (repression of anterior Hox genes by posterior ones: PP) is important in translating temporal to spatial collinearity. We thus demonstrate for the first time that two collinear Hox interactions are important for vertebrate axial patterning. These findings considerably extend and clarify earlier work suggesting the existence and importance of PP and PI, and provide a major new insight into genesis of the body axis.

## Introduction

Understanding the developmental mechanisms mediating embryogenesis and identifying the roles and regulation of their regulatory genes are of key importance for developing and applying key emergent technologies in modern medicine: stem cell therapy, in vitro organoid culture, targeted destruction of specific cancers. Important for all of these approaches are the Hox genes, specifiers of positional identity in the embryo [1–4]. Investigation of these genes [5] opened a new vista in developmental biology and medicine. Here, we reveal how key novel properties of the Hox genes are crucial for their coordinated expression and function.

Hox genes regulate the specification of positional identities along the anteroposterior (A-P) axis during development [6–9]. In most vertebrates, these genes are organised in four clusters (HOXA-D) on different chromosomes. Homologous members of the different clusters have been divided into 13 paralogous groups (HOX1-13) [8, 10]. An intriguing feature of Hox paralogues is that their 3’ to 5’ arrangement on the chromosome matches their temporal expression sequence in development (temporal collinearity) and their spatial order of expression along the vertebrate A-P axis (spatial collinearity) [6, 10]. There is a third form of collinearity: quantitative collinearity, where the amplitude of Hox gene expression correlates with a Hox gene’s 3’to 5’position in a cluster. This occurs only in limb development and is irrelevant for axial patterning. Temporal and spatial collinearities are obviously somehow key to the regulation of the precisely ordered Hox expression in axial patterning. However, both the mechanisms underlying collinearities and the nature of their role in axial patterning are still quite poorly understood. There has been no consensus about the nature of these mechanisms. We present evidence below that collinearity and axial patterning both depend on specific collinear Hox-Hox interactions. These novel findings are important for understanding vertebrate axial patterning.

Hox genes are first expressed, from early gastrulation on, in a temporally collinear order in the non-organiser mesoderm (NOM: i.e., all gastrula mesoderm excluding the organiser and organiser derived tissues) in the *Xenopus* embryo [11] or in its equivalents in the chicken [12] and the mouse [13]. Precise temporal activation of Hox gene expression is crucial for establishing regional identity [14]. For example, an initial delay of *HoxC8* expression results in phenocopies similar to *HoxC8* null mutants [15], suggesting that correctly timed initial expression of Hox genes at earlier stages is crucial for specifying AP identities at later stages. Whereas there are also studies proposing a disconnection between Hox temporal and spatial collinearities [16, 17], our recent studies argue for an indispensable role for Hox temporal collinearity in generating spatial collinearity [11, 18]. Our research in early Xenopus development suggests that the temporally collinear expression of Hox genes serves as a timer during the formation of the A-P axis. This timing information appears to be interpreted and translated into spatial information via a BMP/anti-BMP dependent time-space translation mechanism.

Up until now, however, it is still not clear by which mechanism Hox genes are expressed in a temporally collinear sequence. There are studies correlating temporally collinear Hox expression with progressive 3’to 5’opening of the chromatin, associated with sequential movement of Hox genes from an active to an inactive chromatin compartment [16, 19]. Although this explanation has evidence supporting it, it is not the whole story. Other mechanisms are also involved, since Hox temporal collinearity requires synchronisation of the structurally different Hox clusters within cells and synchrony between different cells in the mesoderm of the gastrula. One possible mechanism involved is collinear Hox interactions within clusters and between different clusters. There are two types of these Hox interactions: posterior prevalence (PP) [20, 21], meaning that 5’ posterior Hox genes dominate more 3’ anterior Hox genes; and posterior induction (PI) [22], meaning that more anterior Hox genes induce the expression of more posterior ones. PP was discovered in the Drosophila embryo and PI in human embryonal carcinoma cells. The importance of PP and PI in these two systems is unclear but both clearly have explicit functions in the early vertebrate embryos where they participate in early patterning of the main A-P body axis. In early *Xenopus* embryos, ectopic expression of *HoxB-4* and *HoxA-7* both repressed expression of more 3’ anterior Hox genes, whereas they induced expression of more 5’ posterior Hox genes [23]. Notably, in these studies, Hox genes also showed autoregulation—inducing their own expression and that of members of their own paralogue groups. Moreover, knocking down the complete *Xenopus* Hox paralogous group 1 (PG1) repressed the expression of the Hox1 paralogues themselves and that of all more posterior genes examined [24], indicating that Hox1 functionality is somehow required for generating Hox spatial collinearity. There is also evidence that some Hox—Hox interactions are paralleled by the interactions between Hox genes and Hox associated microRNAs [25–28]. Taken together, these and other findings suggested that Hox interactions, that can occur at different levels (transcriptional, post-transcriptional, and post translational) [28–32], play a role in driving Hox temporal collinearity and axial patterning. It is notable that because these interactions coordinate Hox behaviour of single cells across tissues, like the NOM, they contribute to a notable fundamental and surprising feature of collinearity namely the interrelation of phenomena spanning over a wide range of spatial dimensions: on one hand the macroscale extent of embryonic ontogeny (up to 1mm) and on the other hand the microscale dimension of a Hox gene cluster (of the order of 100 nm) [33].

The experiments above were necessarily done using either a single Hox gene or paralogue group or a single microRNA or in one case two Hox genes, representing two different paralogue groups. In the present investigation, we used ectopic expression of multiple Hox genes in wild-type and noggin-dorsalised (*Hox*-free) *Xenopus* embryos, to test the generality of and expand our understanding of the findings above. This approach, rather than inactivating multiple Hox genes was chosen to simplify comparing the functions of the different Hox paralogue groups. We ectopically expressed 4 different Hox genes, representing 4 different paralogue groups, active at 4 different axial levels and examined effects of these different treatments on a greater number (totally 16) of different axial position markers, including 3 determinants for different levels in the anterior head as well as 13 Hox genes, using two different analysis methods. We also examined the time of Hox action for Hoxb4, using a time-activatable GR construct. Timed Hoxb4-GR activation by dexamethasone showed that posterior induction occurs by early gastrulation, (St. 10.5), in NOM mesoderm and underlies early temporal collinearity which drives later spatial collinearity and axial patterning. Posterior prevalence starts later (at St. 12–15) and presumably mediates time-space translation in mesoderm and neurectoderm. Hox genes also exerted posterior prevalence over head determinants. Ectopically expressing different Hox genes in axis deficient, Hox deficient dorsalised embryos rescued different predictable parts of the A-P axis and the corresponding different sequences of Hox gene expression. These findings represent important novel insights into vertebrate A-P patterning and they emphasize the importance of interactions between the Hox genes in this process.

## Methods

### Frog husbandry and microinjection

All procedures involving the use of animals for this study were approved by the animal experiments committee (dierexperimentencommissie, DEC) of Leiden university. Frogs (Xenopus laevis) were housed and maintained in a temperature-controlled aquarium. Animal welfare was recorded on a daily basis and the use of animals was reported annually to DEC. Embryos were collected from naturally mated females and staged according to Nieuwkoop and Faber [34]. For Hox ectopic expression, about 250 pg mRNA was injected to each blastomere at 2-cell or 4-cell stage. Dexamethasone (DEX) (Sigma) treatment was carried out for *HoxB-4* GR injection. DEX was added to culture medium at a concentration of 10 μM. Embryos were then incubated in DEX for 2h. For Hox and *Noggin* co-injection, the mRNAs were mixed together before injection and 200 pg and 140 pg were injected respectively to each blastomere at 2-cell or 4-cell stage.

### Quantitative RT-PCR

Total RNA was isolated from three whole embryos using the RNeasy kit (Qiagen), and cDNA was synthesised using the iScript cDNA synthesis kit (Bio-rad). Quantitative RT-PCR was carried out on the CFX96 (Bio-rad) using SYBR green Q-PCR Mater Mix (Bio-rad). The measurements were normalised to *histone H4* and were repeated at least three times. Fold changes were calculated using the 2^-ΔΔCt method. Primers used in this study can be found in S1 Table.

### Whole mount in situ hybridization

Embryos were harvested when they reached the desired stages. Prior to in situ hybridization, they were fixed overnight in MEMFA at 4°C and stored at -20°C in 100% methanol. Whole mount in situ hybridization (WISH) was performed as previously described [11].

### DNA constructs

In situ probes: *Hoxd1*, *Hoxc6*, *Hoxb9*, *Gbx2*, *Otx2* [24], *Six3* [35–37]. Expression constructs: *Hoxd1*[24], *Hoxb4GR*, *Hoxa7*: [23], *Hoxb9*: E. De Robertis, unpublished.

## Results

### Ectopic Hox expression rescues part of the A-P axis and a predictable Hox sequence in noggin-injected embryos

**Fig 1:**
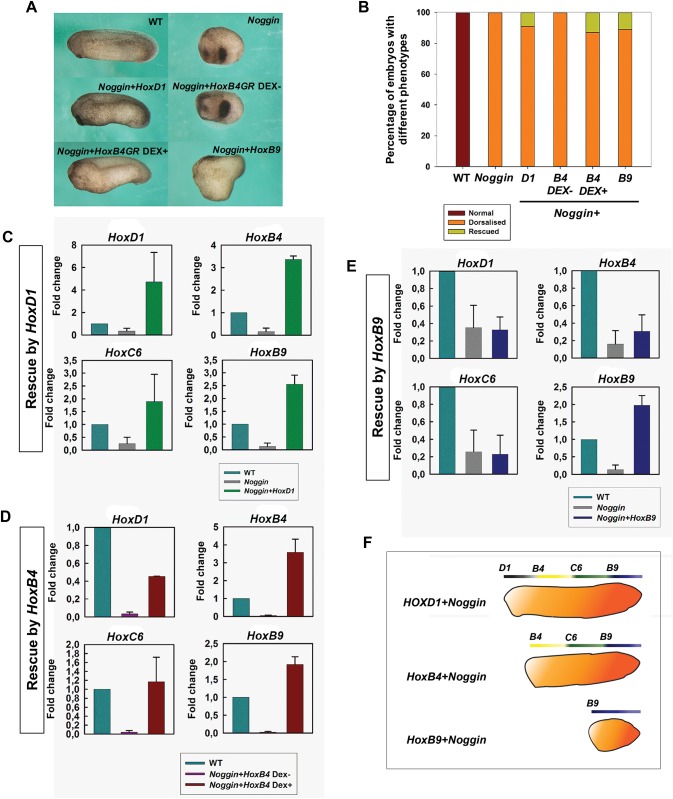
Anteroposterior axis is rescued by ectopic Hox expression in noggin-dorsalised embryos. (A) Morphological phenotypes of embryos in different Hox rescue treatments. Anterior is to the left and dorsal is up. (B) Percentage of embryos showing different phenotypes in different treatment groups. From left to right: wild-type (n = 40); Noggin only (n = 32); Noggin and HoxD-1 co-injection (n = 133); HoxB-4 GR and Noggin co-injection, without Dex treatment (n = 46); HoxB4 and Noggin co-injection, with Dex treatment at st.8 (n = 90); HoxB-9 and Noggin co-injection: n = 140. (C-E) Q-PCR for HoxD-1, HoxB-4, HoxC-6 and HoxB-9 in different rescue groups: rescue by HoxD1 (C), rescue by HoxB4 (D), and rescue by HoxB-9. Data are represented as mean ± SEM. (F) Schematic showing different portions of A-P axis and different Hox genes rescued by HoxD1, B4 and B9 respectively.

To understand how Hox gene expression is regulated during A-P axis formation, we first did ectopic Hox expression in noggin-injected embryos. Noggin mimics the anti-BMP function of the Spemann organiser and gives rise to dorsoanteriorised embryos with no A-P axis [38, 39]. Since initiation of Hox activation is BMP-dependent [40], *noggin*-injected embryos contain little or no endogenous Hox expression and thus create an essentially *Hox*-free environment. We co-injected *noggin* RNA with *HoxD-1*, *HoxB-4* or *HoxB-9* RNAs respectively at the 2- or 4-cell stage. As reported previously, the embryos in the *noggin*-only groups showed a dorsalised phenotype and no axis. Each of the co-injected groups, however, restored a different portion of the A-P axis (Fig 1A). *HoxD-1* injection rescued a long axis and Hoxb4 an intermediate axis, whereas *HoxB-9* injection only rescued a tail. (Fig 1A and 1B).

In the rescued embryos, rescue of phenotype was accompanied by restoration of the relevant Hox gene expression. The expression of four Hox genes, *HoxD-1*, *HoxB-4*, *HoxC-6* and *HoxB-9* was examined (Fig 1C–1E). As previously reported [11], there was no Hox expression (or low levels of expression) in dorsalised embryos (*noggin*-injected). In *HoxD-1-noggin* co-injected embryos, all the four Hox genes examined were rescued (Fig 1C), whereas in *HoxB-4-noggin* co-injected embryos, *HoxB-4*, *HoxC-6*, and *HoxB-9* but not *HoxD-1* were rescued (Fig 1D). *HoxB-9-noggin* co-injection rescued only *HoxB-9* (Fig 1E). These results indicate that the Hox sequence was reinitiated from the injected value (Hox1, Hox4 and Hox9, respectively) (Fig 1F).

### Modulation of Hox expression in wild type embryos systematically perturbs the A-P axis and arrests the endogenous Hox gene expression sequence at predictable points

**Fig 2:**
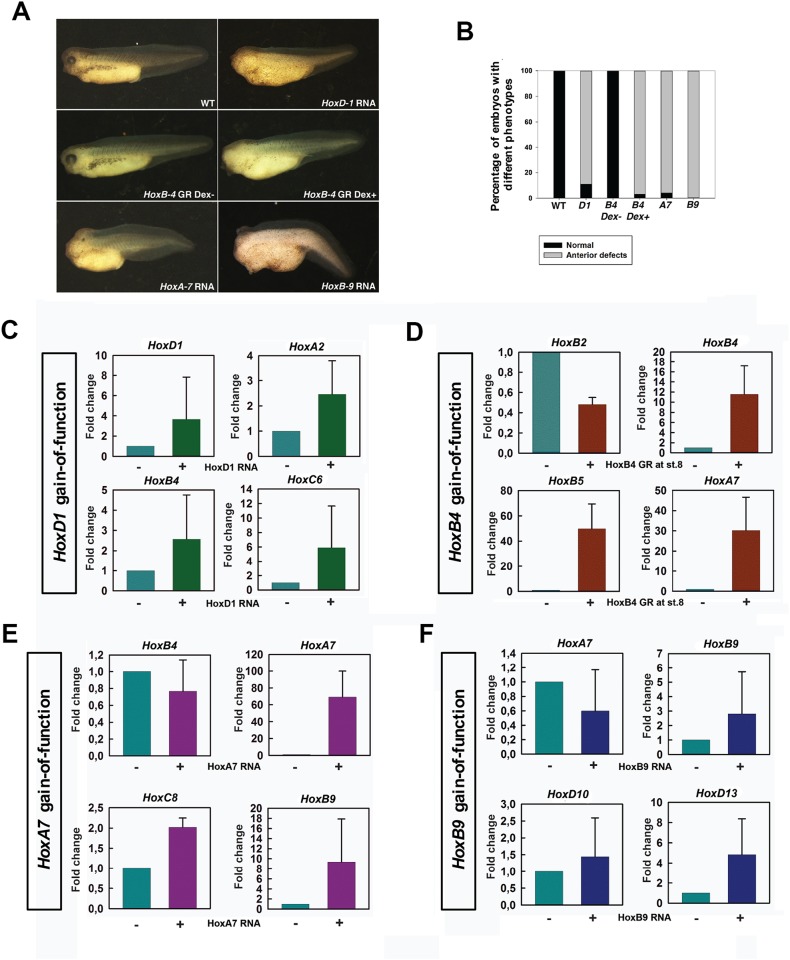
Ectopic Hox expression in wild-type embryos affects axis formation and endogenous Hox expression. (A) Phenotypes of embryos injected with different Hox RNA. (B) Percentage of embryos showing anterior defects. From left to right: wild-type (n = 30), HoxD-1 injected (n = 54), HoxB-4 GR injected (without Dex treatment) (n = 40), HoxB-4 GR injected (with Dex treatment at st.8) (n = 60), HoxA-7 injected (n = 45), HoxB-9 injected (n = 36). (C) Q-PCR for HoxD1, A2, B4 and C6 in HoxD1 injected embryos. (D) Q-PCR for HoxB2, B4, B5 and A7 in HoxB4 GR injected embryos (activated at st.8). (E) Q-PCR for HoxB4, A7, C8 and B9 in HoxA7 injected embryos. (F) Q-PCR for HoxA7, B9, D10 and D13 in HoxB9 injected embryos.

We were interested to know if the Hox regulation observed in *noggin*-injected embryos reflects what happens during normal development. To investigate this, we ectopically expressed *HoxD-1*, *B-4*, *A-7* and *B-9* in WT embryos. As reported previously for *HoxB4* [23], ectopic Hox expression reduced the anterior portion of the A-P axis in most of the embryos from all the four injections (Fig 2A and 2B). Embryos injected with *HoxB9* showed the most severe reduction of the anterior structures, whereas those injected with *HoxD-1* showed the least (Fig 2A). Moreover, it has been known that the precise temporal control of Hox initiation is important for its function [14]. By doing timed activation of *HoxB-4*, we also found that ectopic expression of Hoxb4 before or at rather than after the time of its endogenous expression induced an effect on axis formation (S1 Fig).

To find out what happened in the embryos in Fig 2A, we examined gene expression in these embryos. Consistent with the phenotypes, ectopic Hox gene expression generally repressed the expression of Hox genes anterior to the normal expression position of the ectopic mRNA, while inducing those at the same position or posterior to it (Fig 2C–2F and S2 Fig). For example, in *HoxD-1* injected embryos, *HoxD-1*, *A-2*, B-4, *C-6*, *A7* and *B-9* were induced, showing elevated expression levels and anteriorisation of their expression domains (Fig 2C and S2A Fig). Ectopic expression of *HoxB-4* at stage 8 repressed *HoxD-1*, *B-2* and *D-3*, while inducing *HoxB-4*, *B-5*, *C-6*, *A-7* and *B-9* (Fig 2D and S2B Fig). In *HoxA-7* injected embryos, *HoxD-1* and *B-4* were repressed, while *HoxA-7*, C-*8*, and *B-9* were induced (Fig 2E and S2A Fig). *HoxD-1*, *B-4*, *C-6* and *A-7* were repressed in *HoxB-9* injected embryos, whereas *Hoxb-9*, *D-10*, *C-12*, and *D-13* were induced (Fig 2F and S2C Fig). Notably, *Hoxc-6* (the important Xenopus Hox6 gene for A-P patterning) was not inhibited by *HoxA-7* injection (S2A Fig), suggesting the possibility that the repression of anterior genes by posterior ones does not happen in a cascade manner (i.e. that anterior neighbours are not necessarily (the only) direct targets).

### The interactions also involve more anterior genes

**Fig 3:**
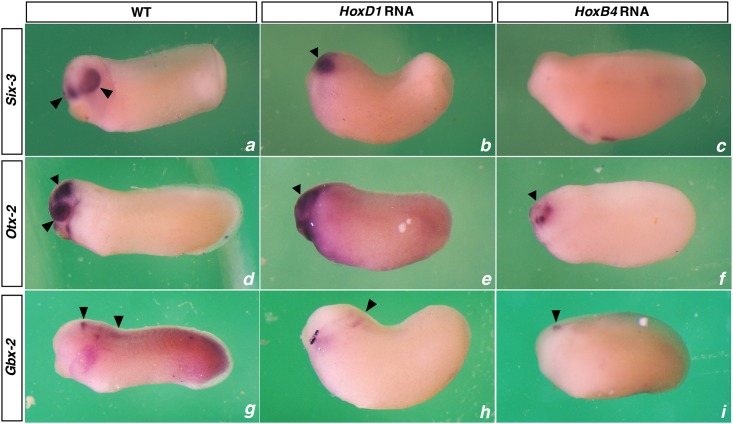
Ectopic Hox expression also affects the expression of anterior head genes. Expression of Six-3 (a, b, c), Otx-2 (d, e, f) and Gbx-2 (g, h, i) are shown for WT, HoxD-1 injected and HoxB-4 GR injected (activated at st.8) embryos.

Since embryos ectopically expressing different Hox genes showed different levels of head defects (Fig 3), it was interesting to know whether or not the expression of anterior head genes are affected. To answer this question, we examined the expression of three anterior genes: *Six-3*, a forebrain marker; *Otx-2*, a forebrain and mid-brain marker; and *Gbx-2*, an anterior hindbrain marker in embryos ectopically expressing *HoxD-1* and *B-4* (at st.8) (Fig 3). Like Hox genes, these anterior genes were also affected by Hox ectopic expression. In both *HoxD-1* and *B-4* injected embryos, the expression of these anterior genes was repressed, with *HoxB-4* injected embryos showing more significant repression.

### Dynamics of Hox interactions

The above results clearly show that the expression of Hox genes is induced by their own expression (auto-regulation) and by that of genes anterior to them (posterior induction) while being inhibited by the expression of genes posterior to them (posterior prevalence). To understand how these Hox interactions occur in detail, a timing experiment was carried out to study the dynamics of Hox interactions. To do this, *HoxB-4* was ectopically expressed at st.8. We then followed the activation of *HoxB-4* and its effects on itself and other Hox genes with time in different tissues.

**Fig 4:**
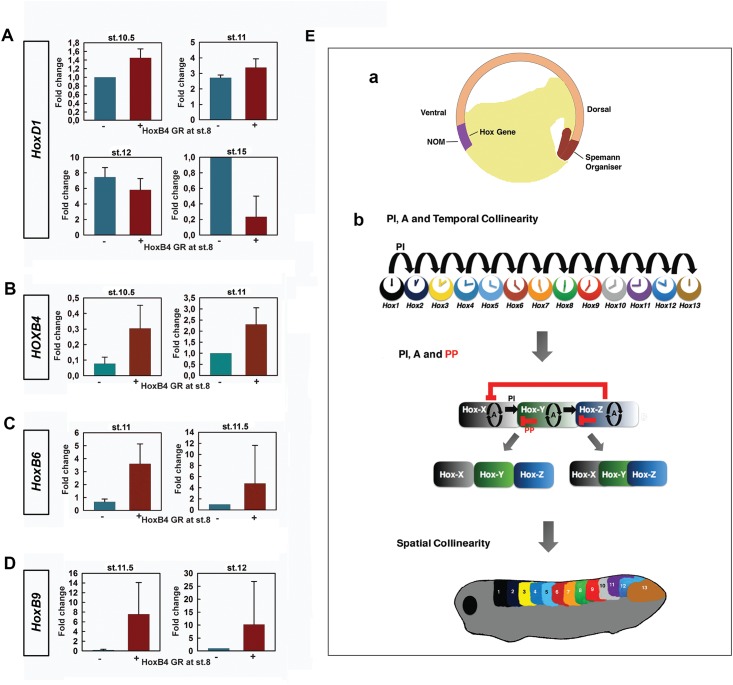
Dynamics of Hox interactions indicates different roles for auto-regulation, posterior induction and posterior prevalence in A-P patterning. (A) Q-PCR for HoxD1 at st.10.5, 11, 12 and 15 in WT and HoxB4 GR (activated at st.8) injected embryos. (B) Q-PCR for HoxB4 at st.10.5 and 11 in WT and HoxB4 injected embryos. (C) Q-PCR for HoxB6 at st.11 and 11.5 in WT and HoxB4 injected embryos. (D) Q-PCR for HoxB9 at st.11.5 and 12 in WT and HoxB4 injected embryos. (E) The known facts concerning auto-regulation, posterior induction and posterior prevalence in A-P Patterning. (a) Hox genes start to be expressed from early gastrulation onward in the non-organiser mesoderm (NOM), where there are high levels of BMP. At this stage, their nested expression domains overlap fully with each other. (b) During gastrulation and early neurulation, auto-regulation (A) and posterior induction (PI) together enable Hox genes (coloured discs) to be expressed in a temporal order that matches their 3' to 5' order on the chromosome (temporal collinearity) The sequential times of initial expression of the neighbouring Hox genes are indicated by the small clock faces. Since the precise control of Hox activation time is vital to function, posterior induction (black arrows) may possibly occur in a cascade manner to ensure the expression of Hox genes in the correct order. Data is not presently available to determine whether this is the case. Starting from neurulation, posterior prevalence (PP) exerts its influence in neurectoderm and paraxial mesoderm, where there are relatively low levels of BMP. The coordination between auto-regulation, posterior induction and posterior prevalence during this stage helps to establish a pre-pattern, resulting in non-overlapping or partially overlapping expression. Notably, posterior prevalence does not happen in a cascade manner since it is not required for driving the Hox timer. Later during axis elongation, these earlier events lead to a spatial pattern being established (spatial collinearity).

Interestingly, *HoxB-4* and *HoxD-1* came on simultaneously in NOM mesoderm at St. 10.5 (Fig 4A and 4B; S3A and S3C Fig), which is earlier than the expression of *HoxB-4* in wild-type embryos (st.11). The advanced expression of *HoxB-4* in injected embryos was followed by *HoxB-6/C-6* and *HoxB-9* at st. 11 and 11.5 respectively (Fig 4C and 4D; S3A and S3C Fig). The expression of these genes in wild-type embryos, however, started at st.11.5 and st.12 respectively (Fig 4C and 4D; S3A and S3C Fig). At all the gastrula stages examined, *HoxD-1* was expressed in injected embryos. It was then turned off in neurectoderm and paraxial mesoderm in some of the embryos at early neurula stage (St. 15) (Fig 4A and S3B Fig), a stage at which the expression of *HoxD-13* is initiated (S4 Fig). These findings suggest specific roles for posterior induction and posterior prevalence in temporal collinearity and axial patterning (Fig 4E).

## Discussion

In this study, we present evidence that collinear Hox-Hox interactions play a significant role in driving the temporally sequential expression of Hox genes during Xenopus embryogenesis. These interactions also involve more anterior genes that specify A-P values in the head. There is much evidence that the vertebrate A-P pattern is generated temporally sequentially from anterior to posterior, with anterior structures being specified early and posterior ones late [41]. Collinear interactions between anterior head genes and Hox genes and among Hox genes discovered in this study are consistent with this phenomenon, and provide a promising explanation for its underlying mechanism.

### Hox gene expression is self-regulated by collinear interactions

One important type of Hox-Hox interaction is posterior induction, referring to induction of posterior Hox genes by anterior ones. It has been shown to be important. For example, by abrogating it. Hox paralogue group 1 knockdown abrogates or compromises expression of all more posterior Hox genes examined [24]. The existence of posterior induction was clearly shown in this study by rescue of the axis in *noggin*-injected embryos, which made a formless mass of tissue containing some head structures but no A-P axis (Fig 1A and 1B). This phenotype involves an inhibition of Hox gene expression in these embryos [11, 42], because the initial expression of Hox genes during gastrulation is BMP-dependent [40, 43]. Since *Noggin*-injected embryos created an environment that is essentially BMP free and *Hox*-deficient, rescue of the Hox sequence in them by *HoxD-1*, *B-4* and *B-9* ectopic expression suggests (Fig 1C–1F) that the Hox timer is self-regulatory. Once it starts ticking, it will keep running until the finish. Notably, since the Hox genes we examined were from different clusters, these results indicate that Hox interactions are able to auto-regulate the expression of paralogous Hox genes, and to coordinate Hox expression across clusters.

### Posterior prevalence and posterior induction occur generally among Hox genes

The above results in *noggin*-injected embryos suggest that posterior induction plays a vital role in driving the Hox timer, which is the key to A-P patterning (Fig 1). Posterior induction was also observed in WT embryos. In our study, ectopic expression of *HoxD-1*, *B-4*, *A-7* and *B-9* in WT embryos induced their own expression and that of paralogues and of more posterior Hox genes (Fig 2 and S2 Fig). The induction of posterior genes by ectopically expressed Hox genes started from the immediate neighbours of the ectopically expressed gene. These results suggest that posterior induction also operates during normal development, and possibly that it works via a cascade.

Another important Hox-Hox interaction is posterior prevalence (PP), which we define here simply as more posterior Hox genes inhibiting action of more anterior Hox genes [44, 45]. Accumulating evidence shows that PP occurs at different molecular levels [28, 29, 31, 32]. Since the only important point to us here is the functional relevance of PP, the level of action and the literature discussing it are not dealt with further in this paper. Similarly as previously reported [23], ectopic expression of a Hox gene imposes corresponding morphological and central nervous system (CNS) defects (Fig 2A and 2B), generally inhibiting formation of structures anterior to its endogenous zone of expression. Molecular analysis showed that ectopic expression of each Hox gene examined inhibited expression of more anterior Hox genes (Fig 2C–2F and S2A–S2C Fig). However, not all anterior genes are always repressed, e.g. *HoxC-6* was still expressed in *HoxA-7* injected embryos (S2A Fig). There is much evidence that axial Hox gene expression zones develop a strong sharp anterior border whereas expression diminishes posteriorly. PP presumably has importance for generating this boundary.

### Collinear interactions also exist between Hox genes and head genes

Another interesting question is whether a similar timing mechanism operates in the specification of more anterior A-P positional values in the head. There is evidence that the homeobox genes *Six-3*, *Otx-2* and *Gbx-2* specify different sequential levels in the head, similarly as the Hox genes do this in the trunk-tail part of the axis. In zebrafish, the expression of *Six-3*, *Otx-2*, *Gbx-1* (the counterpart of *Xenopus Gbx-2*), and *Hoxb1b* is sequentially induced by timed anti-BMP signals from mid-blastula to early gastrula stage [46, 47]. Moreover, there is evidence that Gbx-2 is repressed by *Hoxa2* [48]. Knockdown of the complete Hox paralogue group 1, however, results in a posterior expansion of the *Gbx-2* expression domain [24]. Interactions among these anterior genes have also been reported. For example, ectopic expression of *Gbx2* has been shown to suppress *Otx-2* and *Six-3* [49]. Consistent with these findings, we also found here that ectopic expression of *HoxD-1* and *HoxB-4* repressed the expression of *Six-3*, *Otx-2* and *Gbx-2* (Fig 3). These findings suggest that collinear interactions also exist among these genes and between these genes and the Hox genes. They and the Hox genes seem to constitute an integral sequence for time dependent vertebrate axial patterning [50].

### Posterior prevalence and posterior induction exert their influence at different stages and serve different purposes during A-P patterning

Gain-of-function and rescue experiments together have shown a role for posterior prevalence and posterior induction in establishing the spatial pattern of Hox expression. However, dynamic analysis of Hox gene expression in *HoxB-4-GR* injected WT embryos indicated that these two Hox-Hox interactions operate at different stages. Using a GR construct, we ectopically expressed *HoxB-4* at st.8, long before its initial expression at St.11 during normal development. Interestingly, in this experiment the initial expression of *HoxB-4*, *HoxB6/C6* and *HoxB-9* were brought forward while still keeping their temporal order of expression (Fig 4 and S3 Fig). Since in *HoxB-4* injected embryos the expression of these genes was anteriorised at st. 26 (S2B Fig), these results suggest an association between temporal expression and spatial expression. It is also interesting to note that the endogenous expression of *HoxD-1* was not initially repressed by *HoxB-4*, but started to be turned off at st.15 the stage at which the last paralogue group of Hox genes are first expressed (S4 Fig), suggesting that posterior induction and posterior prevalence function at different stages and in different tissues.

The difference in stages, at which posterior prevalence and posterior induction operate, may have to do with the purposes they serve during A-P patterning. Posterior induction and possibly auto-regulation are needed for keeping the Hox timer ticking. Posterior prevalence is then not needed. In agreement with this idea, the nested Hox expression zones in NOM mesoderm overlap fully during gastrulation [11]. Posterior prevalence becomes necessary in dorsal paraxial mesoderm and neurectoderm where a spatially collinear Hox pattern develops. Its most important role then is presumably to set up a dynamic equilibrium between posterior induction and posterior prevalence which permits the genesis of dynamically metastable Hox expression zones (These concepts are explained in Fig 4E). That these zones have dynamical stability is shown by phenomena like pattern regulation.

## Conclusion

Our study reveals that three Hox-Hox interactions: auto-regulation, posterior induction and posterior prevalence are of key importance during vertebrate axial patterning. Auto-regulation and posterior induction begin with the first Hox expression, in NOM mesoderm, in the gastrula. They are required for driving the Hox timer to tick from start to finish. Posterior prevalence starts later and is presumably involved in converting the Hox time sequence to a dynamically stable axial pattern. In conclusion: the findings above about collinear Hox interactions provide a promising explanation of the mechanism whereby Hox regulation and function underlie vertebrate A-P patterning.

## Supporting information

S1 Table Primers used for Q-PCR. (DOCX)

S1 Fig Timed ectopic expression of *HoxB-4* at different stages using a dexamethasone (dex) inducible glucocorticoid receptor (gr) construct. (A) Phenotypes of embryos. Anterior is to the left and dorsal is up; (B) Percentage of embryos showing anterior defects. From left to right: wild-type(n = 36), without Dex treatment (n = 30), Dex treatment at st.8 (n = 32), Dex treatment at st.10 (n = 36), Dex treatment at st.11 (n = 40), Dex treatment at st.12.5 (n = 32).
(TIF)

S2 Fig Whole Mount In Situ Hybridization (WISH) for different Hox genes after Hox ectopic expression. (A) The expression of *HoxD-1*, *HoxB-4*, *HoxC-6*, *HoxA-7* and *HoxB-9* is shown for WT (a-e), *HoxD-1* injected (f-j) and *HoxA-7* injected (k-o) embryos. White arrows point to the anterior borders of gene expression. In *HoxD-1* injected embryos, all the genes examined were anteriorised (f: n = 8/12; g: n = 10/13; h: n = 9/16; i: n = 9/13; j: n = 10/15). In *HoxA-7* injected embryos, *HoxD-1* (k, n = 5/10), *HoxB-4* (l, n = 6/14) were repressed, *HoxC-6* (m, n = 15/15) was not affected, and *HoxA-7* (n, n = 11/14) and *HoxB-9* (o, n = 14/16) were anteriorised. (B) The expression of *HoxD-1*, *HoxD-3*, *HoxB-4*, *HoxC-6*, *HoxA-7* and *HoxB-9* is shown for WT (a-f) and *HoxB-4* GR (activated at st.8) injected (a’-f’) embryos. White arrows point to the anterior borders of gene expression. In *HoxB-4* injected embryos, the expression of *HoxD-1* (a’, n = 9/17) and *HoxD-3* (b’, n = 9/14) were repressed, whereas the expression of *HoxB-4* (c', n = 9/15), *HoxC-6* (d', n = 10/18), *HoxA-7* (e', n = 8/13) and *HoxB-9* (f', n = 18/25) were anteriorised. (C) The expression of *HoxD-1*, *HoxB-4*, *HoxC-6*, *HoxA-7*, *HoxB-9* and *HoxC-12* is shown for WT (g-l) and *HoxB-9* injected (g’-l’) embryos. In *HoxB-9* injected embryos, the expression of *HoxD-1* (g’, n = 4/9), *HoxB-4* (h’, n = 11/12), *HoxC-6* (I’, n = 14/14) and *HoxA-7* (j’, n = 6/14) were repressed, whereas the expression of *HoxB-9* (k’, n = 12/12) and *HoxC-12* (I’, n = 9/10) were anteriorised.
(TIF)

S3 Fig Dynamic expression of different Hox genes in *HoxB-4GR* injected embryos. (A) WISH for the expression of *HoxD-1* (a-d and a’-d’), *HoxB-4* (e-h and e’-h’), *HoxC-6* (i-l and i’-l’) and *HoxB-9* (m-p and m’-p’) in WT and *HoxB4GR* (activated at st.8) injected embryos. In both WT (a-d) and *HoxB-4GR* injected embryos (a’-d’), the expression of *HoxD-1* was detected from st.10.5 to st.12. However, the expression of *HoxB-4*, *HoxC-6* and *HoxB-9* were detectable from st.10.5 (e’, n = 5/9), st.11 (j’, n = 7/11) and st.11.5 (o’, n = 5/8) respectively, whereas their endogenous expression started from st.11 (f), st.11.5 (k) and st.12 (p), respectively. (B) WISH for *HoxD-1* expression at st.15 in WT (q) and *HoxB-4GR* injected (q’, n = 4/7) embryos. (C) Schematic showing dynamic expression of HoxD-1, HoxB-4, HoxC-6 and HoxB-9 in WT and *HoxB-4GR* injected embryos.
(TIF)

S4 Fig The expression of HoxD-13 at different stages. The expression of HoxD-13 was examined at st.10, 10.5, 11, 11.5, 12, 13 and 15. It started to be expressed at st.15.
(TIF)
