# Supplementary material for: Collinear Hox-Hox interactions are involved in patterning the vertebrate anteroposterior (A-P) axis
Source: PLoS One. 2017 Apr 11;12(4):e0175287. doi: 10.1371/journal.pone.0175287 (PMC5388487; doi:10.1371/journal.pone.0175287)
Supplement: S1 Table — (DOCX) [file pone.0175287.s001.docx]

| **Gene name** | **Forward primer (5’ to 3’)** | **Reverse primer (5’ to 3’)** |
| --- | --- | --- |
| *HoxD-1* | ACACTTCTTGCGGGGATGTT | AAGTGCTGGAGACTGGTGTG |
| *HoxD-1* 3’ UTR | AAAGTCCTGTAGCCAGACACC | GGATTTGAGGCACAGGGAAC |
| *HoxA-2* | *GGTTACTACAGCCTGCCTCA* | *TTTGACCTGCCTTTCGGTCA* |
| *HoxB-4* | ACTTGTCCCAGGCGAGAAAG | AAGTGGAAGTGGCCTTGGAG |
| *HoxB-4* 3’UTR | CTGCGGTACAAAGGCTGAACCT | CAGGCCCCAAACTGTGTGATC |
| *HoxB-5* | CCGAGACTGACGAATCCACC | TTCCCATCTGGACCTGCCAT |
| *HoxB-6* | AGGAGCAAGACGAGGCAAAG | GGTGTAGGTCTGTCTCCCTCT |
| *HoxC-6* | CCAGGACAAGGACATGCTCAC | TCCAGCTCCAGAGTTTGGTAAC |
| *HoxA-7* | ATTCCGCTGCTCTGCAATGA | CTCCTCCTGCGGGTTAGGTA |
| *HoxA-7 3’ UTR* | TATGGGGTTTGCACGTGACA | AACCCTTTGCTGACTCCTGG |
| *HoxC-8* | CGTCTCCCAGTCTCATGTTCC | CTTGCCTCTCAGTCAGTCCC |
| *HoxB-9* | GACACTGACCGGACTCATCA | TGACTTGTCTCTCGCTCAGG |
| *HoxB-9 3’ UTR* | CTGGAACCAGCAGACTCTCG | CACTTGGCACAGGGAACACA |
| *HoxD-10* | CTGGCTGAGGTGTCTGTGTC | GCTTGTTGGGGTATCGGACT |
| *HoxD-13* | CTGGAACGGGCAGGTTTATT | CACACATATCCGCCTGGTTTAG |
| *Histone H4* | CGGGATAACATTCAGGGTATCACT | ATCCATGGCGGTAACTGTCTTCCT |
