# Supplementary figures and images for: Collinear Hox-Hox interactions are involved in patterning the vertebrate anteroposterior (A-P) axis
Source: PLoS One. 2017 Apr 11;12(4):e0175287. doi: 10.1371/journal.pone.0175287 (PMC5388487; doi:10.1371/journal.pone.0175287)

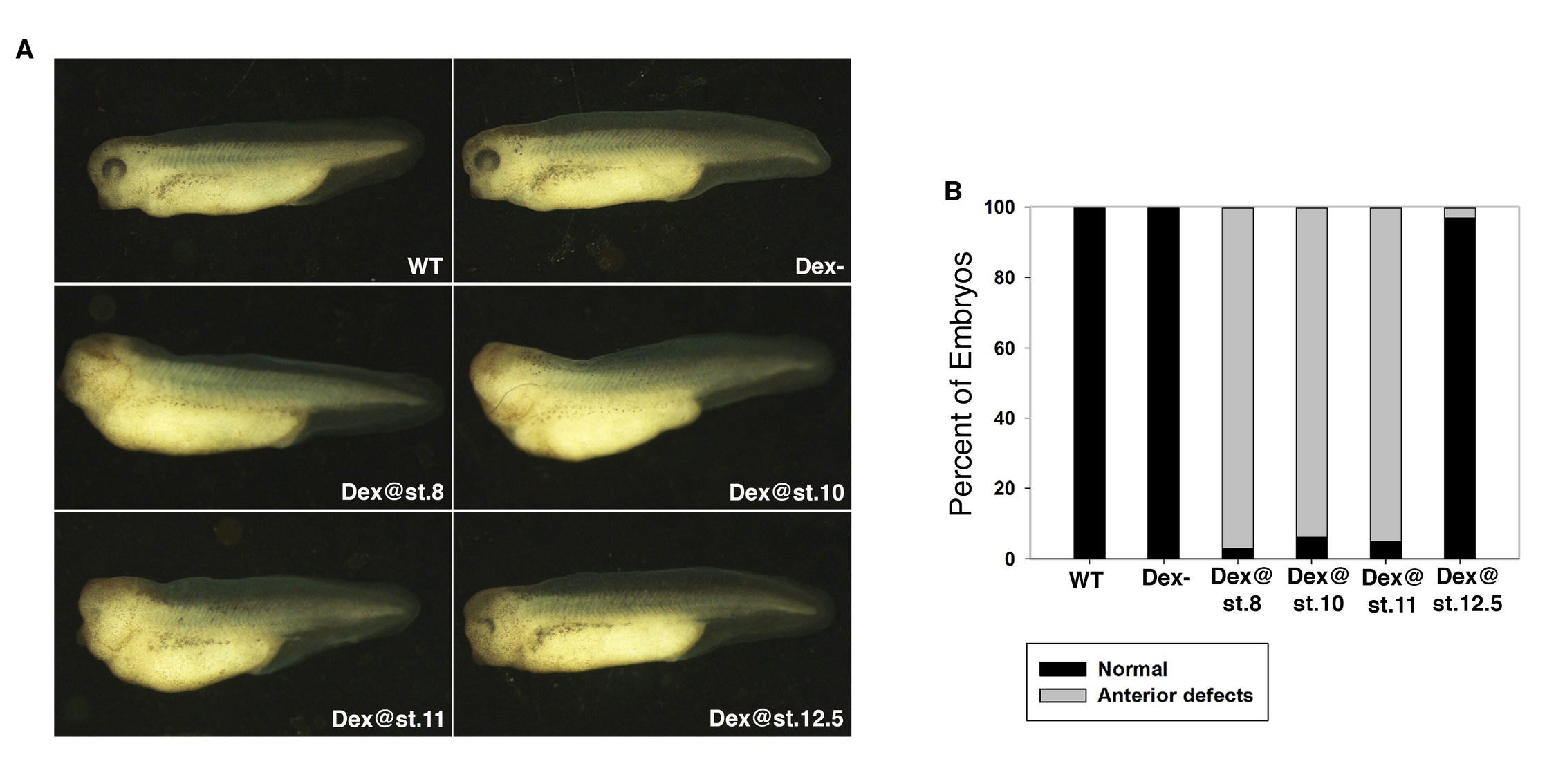

Supplement: S1 Fig — (A) Phenotypes of embryos. Anterior is to the left and dorsal is up; (B) Percentage of embryos showing anterior defects. From left to right: wild-type(n = 36), without Dex treatment (n = 30), Dex treatment at st.8 (n = 32), Dex treatment at st.10 (n = 36), Dex treatment at st.11 (n = 40), Dex treatment at st.12.5 (n = 32). (TIF) [file pone.0175287.s002.tif]

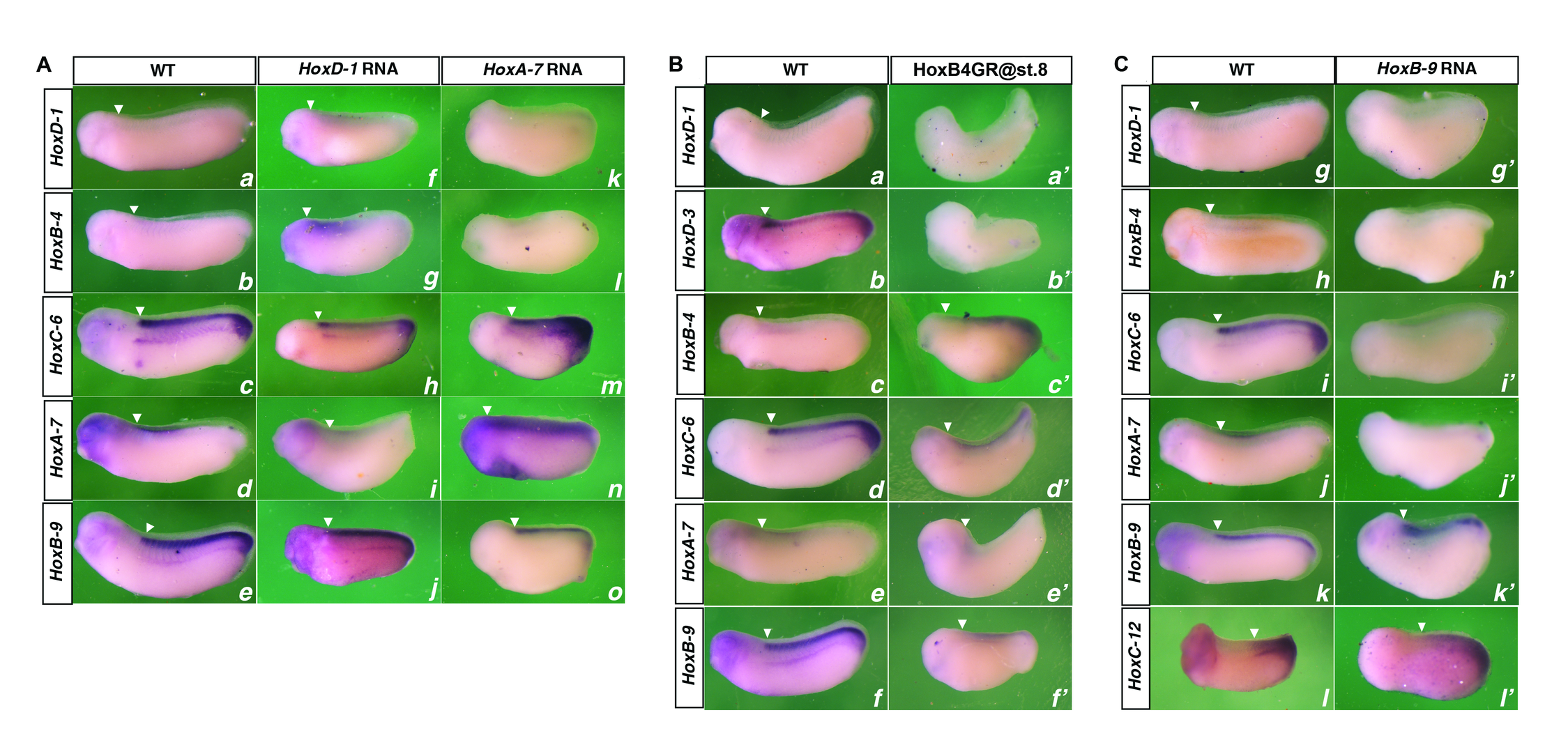

Supplement: S2 Fig — (A) The expression of HoxD-1, HoxB-4, HoxC-6, HoxA-7 and HoxB-9 is shown for WT (a-e), HoxD-1 injected (f-j) and HoxA-7 injected (k-o) embryos. White arrows point to the anterior borders of gene expression. In HoxD-1 injected embryos, all the genes examined were anteriorised (f: n = 8/12; g: n = 10/13; h: n = 9/16; i: n = 9/13; j: n = 10/15). In HoxA-7 injected embryos, HoxD-1 (k, n = 5/10), HoxB-4 (l, n = 6/14) were repressed, HoxC-6 (m, n = 15/15) was not affected, and HoxA-7 (n, n = 11/14) and HoxB-9 (o, n = 14/16) were anteriorised. (B) The expression of HoxD-1, HoxD-3, HoxB-4, HoxC-6, HoxA-7 and HoxB-9 is shown for WT (a-f) and HoxB-4 GR (activated at st.8) injected (a’-f’) embryos. White arrows point to the anterior borders of gene expression. In HoxB-4 injected embryos, the expression of HoxD-1 (a’, n = 9/17) and HoxD-3 (b’, n = 9/14) were repressed, whereas the expression of HoxB-4 (c', n = 9/15), HoxC-6 (d', n = 10/18), HoxA-7 (e', n = 8/13) and HoxB-9 (f', n = 18/25) were anteriorised. (C) The expression of HoxD-1, HoxB-4, HoxC-6, HoxA-7, HoxB-9 and HoxC-12 is shown for WT (g-l) and HoxB-9 injected (g’-l’) embryos. In HoxB-9 injected embryos, the expression of HoxD-1 (g’, n = 4/9), HoxB-4 (h’, n = 11/12), HoxC-6 (I’, n = 14/14) and HoxA-7 (j’, n = 6/14) were repressed, whereas the expression of HoxB-9 (k’, n = 12/12) and HoxC-12 (I’, n = 9/10) were anteriorised. (TIF) [file pone.0175287.s003.tif]

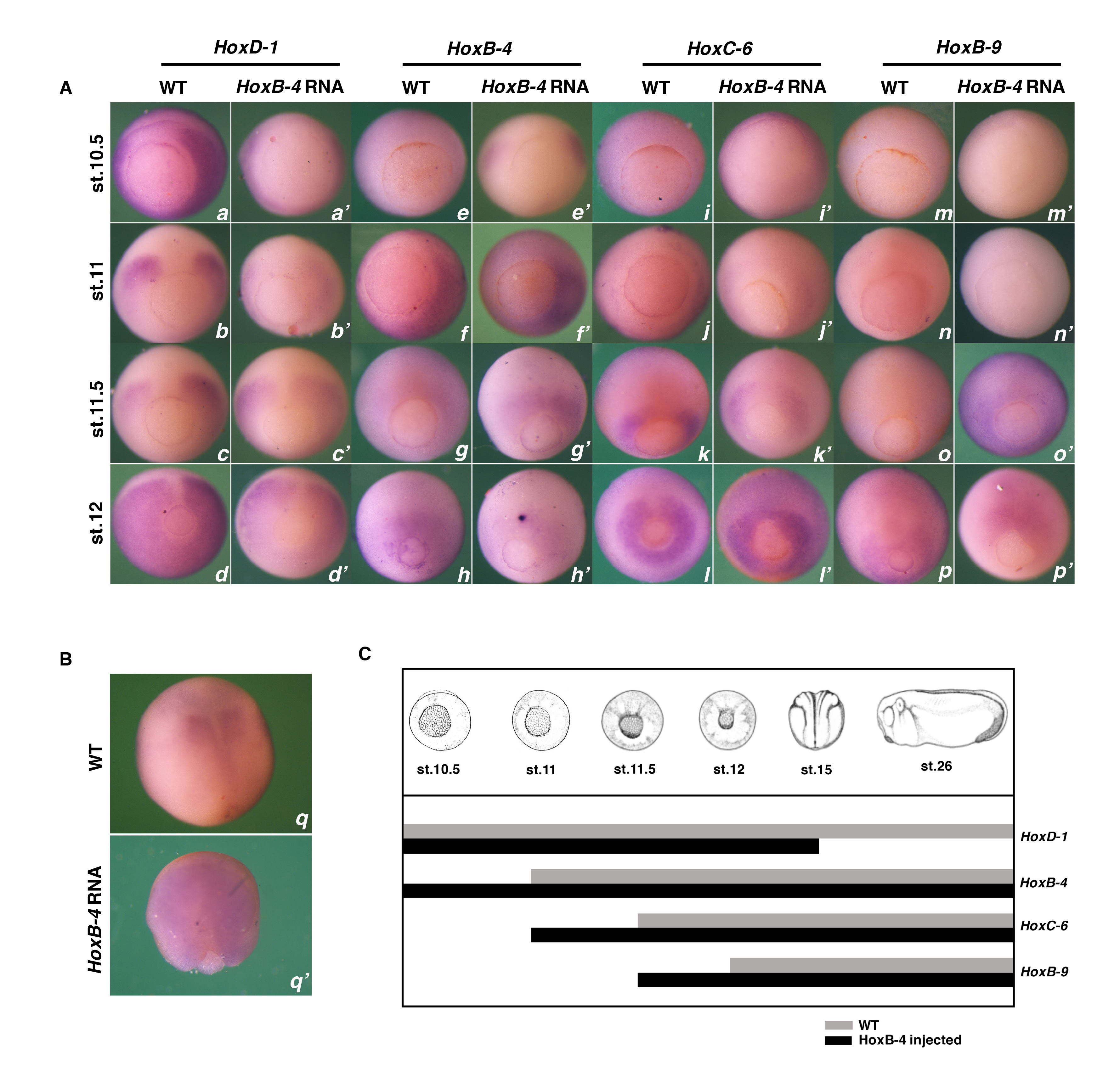

Supplement: S3 Fig — (A) WISH for the expression of HoxD-1 (a-d and a’-d’), HoxB-4 (e-h and e’-h’), HoxC-6 (i-l and i’-l’) and HoxB-9 (m-p and m’-p’) in WT and HoxB4GR (activated at st.8) injected embryos. In both WT (a-d) and HoxB-4GR injected embryos (a’-d’), the expression of HoxD-1 was detected from st.10.5 to st.12. However, the expression of HoxB-4, HoxC-6 and HoxB-9 were detectable from st.10.5 (e’, n = 5/9), st.11 (j’, n = 7/11) and st.11.5 (o’, n = 5/8) respectively, whereas their endogenous expression started from st.11 (f), st.11.5 (k) and st.12 (p), respectively. (B) WISH for HoxD-1 expression at st.15 in WT (q) and HoxB-4GR injected (q’, n = 4/7) embryos. (C) Schematic showing dynamic expression of HoxD-1, HoxB-4, HoxC-6 and HoxB-9 in WT and HoxB-4GR injected embryos. (TIF) [file pone.0175287.s004.tif]

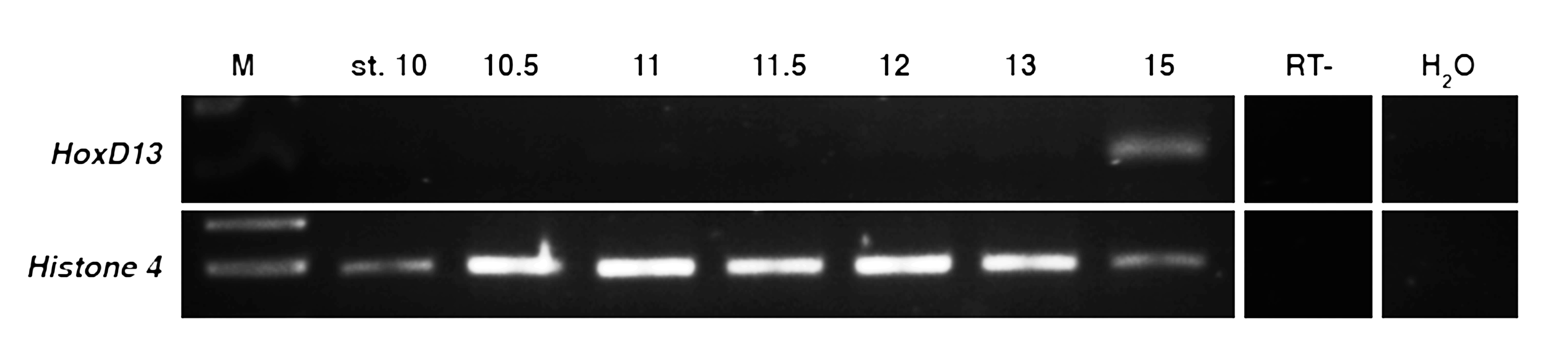

Supplement: S4 Fig — The expression of HoxD-13 was examined at st.10, 10.5, 11, 11.5, 12, 13 and 15. It started to be expressed at st.15. (TIF) [file pone.0175287.s005.tif]
